# Treadmill Exercise in Rats Attenuates Chronic Unpredictable Stress-Induced Depressive-like Behavior Associated with Melatonin–SIRT1/Nrf2 Signaling in the Prefrontal Cortex

**DOI:** 10.33549/physiolres.935743

**Published:** 2026-08-01

**Authors:** Ji-Ping HE, Xiao-Yun SU, Jie MEN, Jian-Mei CUI, Li-Na ZHANG

**Affiliations:** 1 Department of Anatomy, Shanxi University of Medicine, Shanxi, China; 2 Department of Nursing, Shanxi University of Medicine, Shanxi, China; 3 Department of Sports, Shanxi University of Medicine, Shanxi, China; 4 Department of Sports, North University of China, Shanxi, China; 5 Department of Psychiatry, Shanxi province Fenyang hospital, Shanxi, China.

**Keywords:** Treadmill exercise, Chronic unpredictable mild stress, Depressive behavior, Melatonin, Oxidative stress, SIRT1/Nrf2

## Abstract

To explore the ameliorating effect of treadmill exercise on depression-like behavior in rats with chronic unpredictable stress (CUS). Thirty-six Sprague-Dawley rats were divided into three groups (n=12 per group): a control group, a CUS group and a CUS+exercise (CUS+EX) group. The CUS and CUS+EX groups received a 4-week CUS treatment, and the CUS+EX group began a 4-week moderate-intensity treadmill exercise at the second week of CUS treatment. Following the CUS treatment and treadmill exercise, the depressive-like behavior of rats was assessed using the open field test (OFT) and tail suspension test (TST). Plasma melatonin levels were measured using an enzyme-linked immunosorbent assay, and the superoxide dismutase (SOD) activity and malondialdehyde (MDA) levels in the prefrontal cortex (PFC) were detected using commercial kits. Compared with the control group, the CUS group showed shortened latency to first immobility and prolonged total immobility time in the TST, and decreased time spent in the center and reduced crossing and vertical activities in the OFT. Additionally, the SOD activity and the protein expressions of sirtuin 1 (SIRT1) and nuclear factor E2 associated factor 2 (Nrf2) in the PFC decreased, whereas the MDA level increased in the CUS group. Four weeks of concurrent treadmill exercise attenuated the effects of CUS: compared with the CUS group, the CUS+EX group exhibited reduced depressive-like behaviors, prevention of the CUS-induced decline in PFC SOD activity and SIRT1/Nrf2 protein expression and attenuation of the CUS-induced increase in PFC MDA levels. Treadmill exercise concurrently increased plasma melatonin levels and activated the SIRT1/Nrf2 antioxidant pathway in the PFC, which may contribute to the observed improvement in depressive-like behavior.

## Introduction

Depression is a common mental disorder characterized by persistent low mood, anhedonia, feelings of guilt, decreased cognitive function, recurrent thoughts of death and even suicidal behavior, significantly impacting quality of life and increasing the global burden of disease [1].

Approximately 350 million people worldwide suffer from depression, and the prevalence and lifetime risk are around 12.9 % and 10.8 %, respectively [2,3]. Current antidepressants, such as selective serotonin and norepinephrine reuptake inhibitors, are only effective in around 50 %–70 % of patients with major depression and can cause major side effects that lead to early termination of treatment [4]. Therefore, it is important to explore the pathogenesis of depression.

Oxidative stress is considered one of the key pathogenic mechanisms underlying depression [5]. Oxidative stress refers to an imbalance between the production of free radicals in the body and the antioxidant defence system, leading to cell and tissue damage [6]. Studies have shown that levels of oxidative stress are significantly elevated in patients with depression and in animal models, suggesting that oxidative stress plays a key role in the pathophysiological processes of depression [7–9]. In addition, melatonin acts as an important endogenous antioxidant that not only regulates circadian rhythms but also has anti-inflammatory and neuroprotective effects [10,11]. Clinical studies have reported that nocturnal melatonin levels are significantly reduced in patients with depression, suggesting that melatonin may be closely associated with the onset and progression of depressive disorders.

The sirtuin 1 (SIRT1)/nuclear factor E2 associated factor 2 (Nrf2) signaling pathway plays an important role in antioxidant defence and neuroprotection [12]. Specifically, SIRT1 is an NAD-dependent deacetylase that can enhance the antioxidant defence system and reduce oxidative stress damage by activating Nrf2 and its downstream targets [13]. Studies have shown that melatonin can protect the brain from oxidative stress by up-regulating SIRT1 expression and activating the SIRT1/Nrf2 signaling pathway. In addition, as a non-pharmacological intervention, exercise has been shown to relieve depressive symptoms with comparable efficacy to medication [5]. Exercise can not only regulate melatonin levels but also reduce oxidative stress by activating the SIRT1/Nrf2 signaling pathway, thus playing an antidepressant role [14].

However, whether long-term exercise can improve chronic stress-induced depression-like behavior by regulating melatonin levels and activating the SIRT1/Nrf2 signaling pathway needs further investigation. Therefore, this study investigates the effects of 4-week moderate-intensity treadmill exercise on depression-like behavior in rats with chronic unpredictable stress (CUS) and reveals the potential mechanism by which melatonin improves depressive behavior by modulating SIRT1/Nrf2 signaling pathways in the prefrontal cortex (PFC).

We measured plasma melatonin and PFC oxidative stress markers (superoxide dismutase [SOD] activity and malondialdehyde [MDA] level) to capture complementary aspects of circadian regulation and redox state. Melatonin is a key regulator of circadian rhythms and exerts antioxidant and neuroprotective effects; alterations in melatonin signaling have been implicated in mood disorders. Superoxide dismutase activity reflects enzymatic antioxidant defence, whereas MDA is a widely used marker of lipid peroxidation and oxidative damage. Given the established role of the SIRT1/Nrf2 axis in regulating antioxidant responses, these markers were selected to link behavioral phenotypes with underlying redox-related molecular pathways.

## Materials and Methods

### Experimental animals

Male 8-week-old Sprague-Dawley rats (weighing 200±20 g) were provided by the Experimental Animal Center of Shanxi Medical University. The rats were housed in a suitable laboratory environment at 22 °C±2 °C, 55 %±10 % humidity, and a 12-h light/dark cycle (except for reversed day/night cycles during stress procedures). They were allowed to adapt to the laboratory conditions for 1 week before the experiment. Following adaptation, the rats were randomly divided into three groups: the control group, the CUS group and the CUS+exercise (CUS+EX) group, with 12 rats in each group. All experimental procedures were approved by the Ethics Committee of Fenyang College of Shanxi Medical University and conducted according to the NIH Guide for the Care and Use of Laboratory Animals. The experimental procedures in this study were approved by the ethics committee (Approval Number:2025029) on [February 2nd, 2025].

### Chronic unpredictable stress protocol

After 1 week of adaptation, the CUS and CUS+EX groups were subjected to 7 different types of unpredictable stressors daily for 4 consecutive weeks. The same type of stressor did not appear consecutively to ensure unpredictability. Specific stimulation methods included 24-hour fasting, no water for 12 hours, cold stress for 30 minutes (+6 °C), soft non-invasive tail clips (1 minute; side of tail 2 cm from tip), hot plate test (1 minute, T = 50 °C), mixed cage animals (two ‘new’ rats and two ‘old’ rats in the cage) and light/dark cycle reversal (light on from 20.00 to 8.00, light off from 8.00 to 20.00). The control group was not subjected to any interference, except for routine cage cleaning [15,16].

### Treadmill exercise protocol

Rats in the CUS+EX group underwent a moderate-intensity treadmill exercise protocol for 4 weeks. Exercise began at week 2 of the CUS procedure and continued for 4 consecutive weeks. Animals were exercised 6 days per week, 60 minutes per day. Each session consisted of a 5-minute warm-up at 10 m·min^−1^, 50 min at 20 m·min^−1^ (main training) and a 5-minute cool-down at 10 m·min^−1^. The treadmill slope was set to 0°. To reduce potential circadian influences on melatonin and related measures, treadmill sessions were performed consistently in the morning (approximately 09:00–11:00). No electrical shock was used to force running; animals were encouraged gently when necessary. The running speed was gradually increased during the first training week from 10 m·min^−1^ to the target 20 m·min^−1^, which was then maintained for the remaining 3 weeks. This protocol follows previously published moderate-intensity treadmill paradigms in adult rats [17,18].

### Light reversal and sampling interval

The light/dark cycle reversal stressor (light on from 20:00 to 08:00, light off from 08:00 to 20:00) was applied intermittently during the 4-week CUS protocol (typically 1–2 times per week, never on consecutive days). Blood sampling for melatonin measurement occurred at least 48 hours after the final light reversal exposure to allow circadian re-entrainment and avoid acute phase-shifting effects. This interval was chosen to capture stable, chronic melatonin adaptations rather than immediate stress-induced fluctuations.

### Behavioral tests

Behavioral tests were performed following CUS treatment and treadmill exercise.

#### - Open field test

An open field test (OFT) is an experimental method used to evaluate the spontaneous movement and exploratory behavior of animals. Rats were placed in a box (100×100×50 cm) with a grid floor (16 squares, 25×25 cm) under consistent illumination (40 lux, as measured at the centre of the arena) and allowed to move freely for a 3-minute habituation period (not recorded), followed by 5 minutes of recorded activity. This procedure was adopted to minimise stress-induced initial freezing and allow animals to acclimate to the novel environment, thereby capturing more stable exploratory behavior [19]. Parameters for the recorded 5-minute period included horizontal crossings, vertical rearing and time spent in the centre. The box was cleaned with 75 % alcohol after each test.

#### - Tail suspension test

The tail suspension test (TST) is an experimental model used to assess feelings of hopelessness and helplessness in animals. Rats were suspended by their tails from a stand 50 cm above the ground for 6 minutes. The latency to first immobility and total immobility time during the last 4 minutes were recorded [20].

### Anesthesia and blood collection procedure

Prior to decapitation or blood sampling, rats were deeply anesthetized with 3 % pentobarbital sodium (0.01 mL/g, ip) and confirmed unconscious via toe-pinch reflex absence. For tail vein puncture, anesthetized animals were placed in lateral recumbency with the tail extended and warmed (40 °C water bath, 30 seconds) to enhance vasodilation. A 23-gauge needle was inserted into the lateral tail vein approximately 2–3 cm from the tail tip, and 1–2 mL blood was collected into EDTA-coated tubes. This procedure was performed under 150 lux illumination between 8:00 and 10:00 to standardize circadian conditions.

### Enzyme-linked immunosorbent assay for plasma melatonin levels

Plasma melatonin levels were measured using an enzyme-linked immunosorbent assay (ELISA) following behavioral tests. All rats were anesthetized with 3 % pentobarbital sodium (0.01 mL/g, ip) prior to blood collection. Blood samples were collected via tail vein puncture under anesthesia between 8:00 and 10:00 under consistent illumination (150 lux) and centrifuged at 4 °C for 20 minutes at 3,000 rpm, and the supernatant was analyzed. This timing was selected to standardize sampling relative to the light–dark cycle and minimize circadian variation in melatonin levels. A plasma melatonin ELISA kit was purchased from Hangzhou Grans Biotechnology Co., Ltd. (Hangzhou, China). The SOD and MDA assay kits were obtained from Shenyang Wanhe Biotechnology Co., Ltd. (Shenyang, China).

### Measurement of superoxide dismutase activity and malondialdehyde levels in the prefrontal cortex

Following blood collection, six rats from each group were decapitated, and the PFC was rapidly dissected on ice, homogenized to 10 % tissue homogenate and centrifuged at 4 °C for 10 minutes at 4,000 rpm. The supernatant was used to measure SOD activity using the xanthine oxidase method and MDA levels using the thiobarbituric acid assay.

### Western blot analysis of SIRT1 and Nrf2 protein expression in the prefrontal cortex

The remaining rats were decapitated, and the PFC was dissected, homogenized in lysis buffer and incubated on ice for 30 minutes. The lysates were centrifuged at 4 °C for 20 minutes at 12,000 rpm, and the supernatant was used for protein quantification and Western blot analysis. Primary antibodies used were as follows: rabbit anti-SIRT1 polyclonal antibody (1:1000; Cell Signaling Technology, Danvers, MA, USA; Catalog No.2028), rabbit anti-Nrf2 monoclonal antibody (1:1000; Cell Signaling Technology, Danvers, MA, USA; Catalog No. 12721), and rabbit anti-β-actin monoclonal antibody (1:1000; Cell Signaling Technology, Danvers, MA, USA; Catalog No. 8457).

Secondary antibodies: goat anti-rabbit IgG (1:5000; Proteintech, Wuhan, CHN; Catalog No. B900210) and goat anti-mouse IgG (1:5000; Proteintech, Wuhan, CHN; Catalog No. 66009-1-Ig) were used for detection of primary antibodies. Protein bands were visualized using enhanced chemiluminescence (ECL) reagents (Millipore, Billerica, MA, USA) and quantified by densitometric analysis using ImageJ software (National Institutes of Health, Bethesda, MD, USA).

### Statistical analysis

All data are presented as mean±SD. Statistical analyses were performed using SPSS 26.0 (IBM). Data distributions were tested for normality using the Shapiro–Wilk test and for homogeneity of variances via Levene’s test. For datasets that satisfied parametric assumptions, comparisons among groups were performed using one-way ANOVA followed by Tukey’s post hoc test. For datasets that did not satisfy assumptions, the nonparametric Kruskal–Wallis test with appropriate pairwise comparisons were used. All tests were two-tailed, with p<0.05 considered statistically significant. The specific statistical test used for each panel is indicated in the corresponding figure legend.

## Results

### Tail suspension test results

**Fig. 1:**
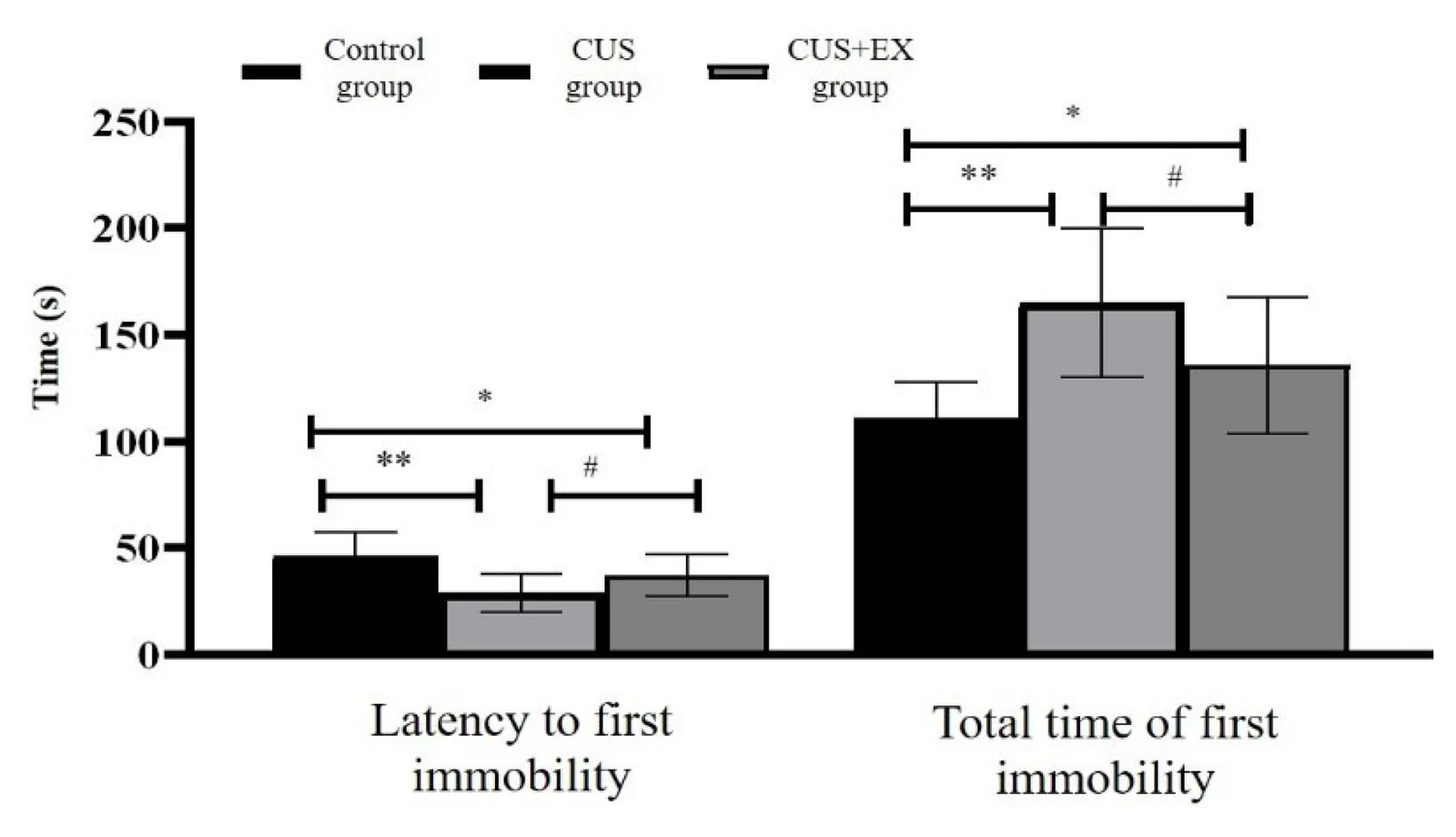
Comparison of the latency to first immobility and the total time of immobility in TST in each group of rats. (n=12). Data are presented as mean ± standard deviation (SD). n=12 per group. Statistical significance: **P<0.01, *P<0.05 vs control group; #P<0.05, CUS+EX group vs CUS group.

Compared with the control group, the CUS and CUS+EX groups showed significantly shorter latency to first immobility and longer total immobility time in the OFT (p<0.01, p<0.05). After 4 weeks of treadmill exercise, the CUS+EX group showed significantly longer latency to first immobility and shorter total immobility time (p<0.05). These results indicate that concurrent treadmill exercise prevented/attenuated the CUS-induced increase in behavioral despair (Fig. 1).

### Open field test results

**Fig. 2:**
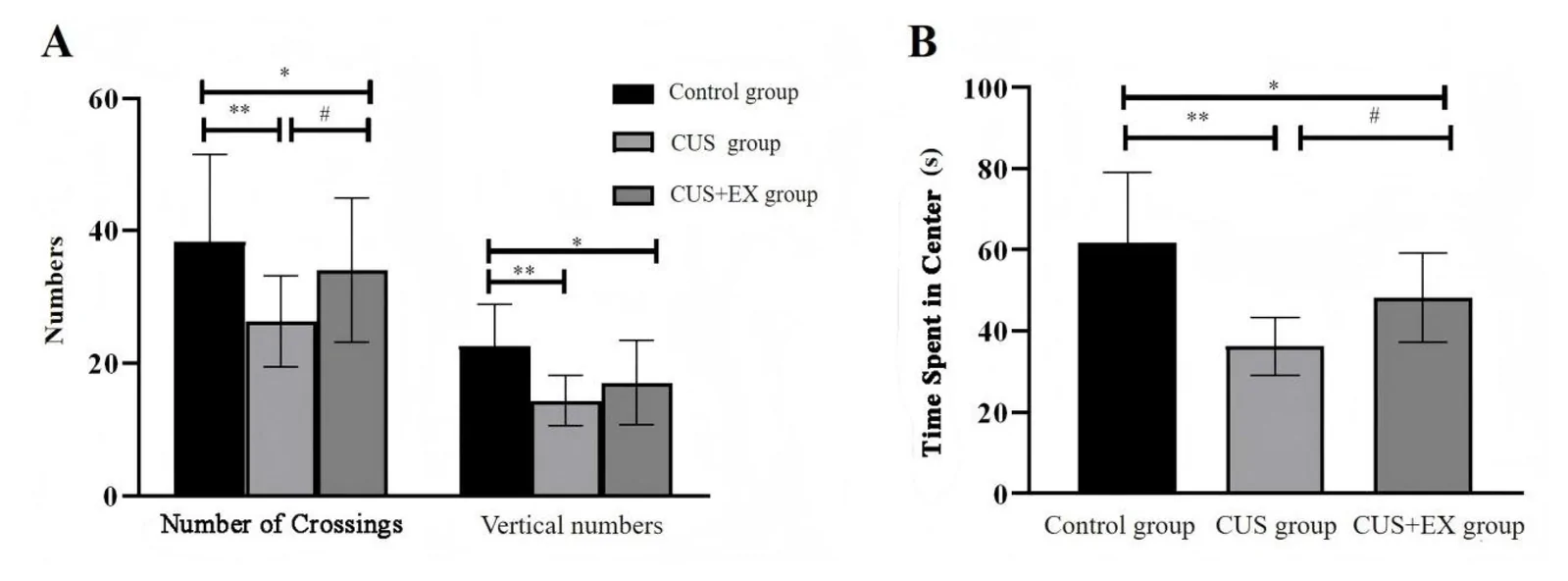
**A,B)** Comparison of the crossing and vertical activity and the time of staying in the center in OFT in each group of rats. Data are presented as mean ± standard deviation (SD). n=12 per group. Statistical significance: **P<0.01, *P<0.05 vs control group; #P<0.05, CUS+EX group vs CUS group.

Compared with the control group, the CUS group had significantly fewer crossings and vertical rearings and spent less time in the center in the OFT (p<0.01, p<0.05). The CUS+EX group likewise differed from the control group in some measures, but compared with the CUS group, the CUS+EX group showed significantly more crossings and a longer time spent in the center (p<0.05), whereas vertical rearing did not differ significantly (p>0.05). These findings indicate that treadmill exercise prevented the CUS-induced decline in exploratory behavior (Fig. 2).

### Plasma melatonin levels and oxidative stress in the prefrontal cortex

**Fig. 3:**
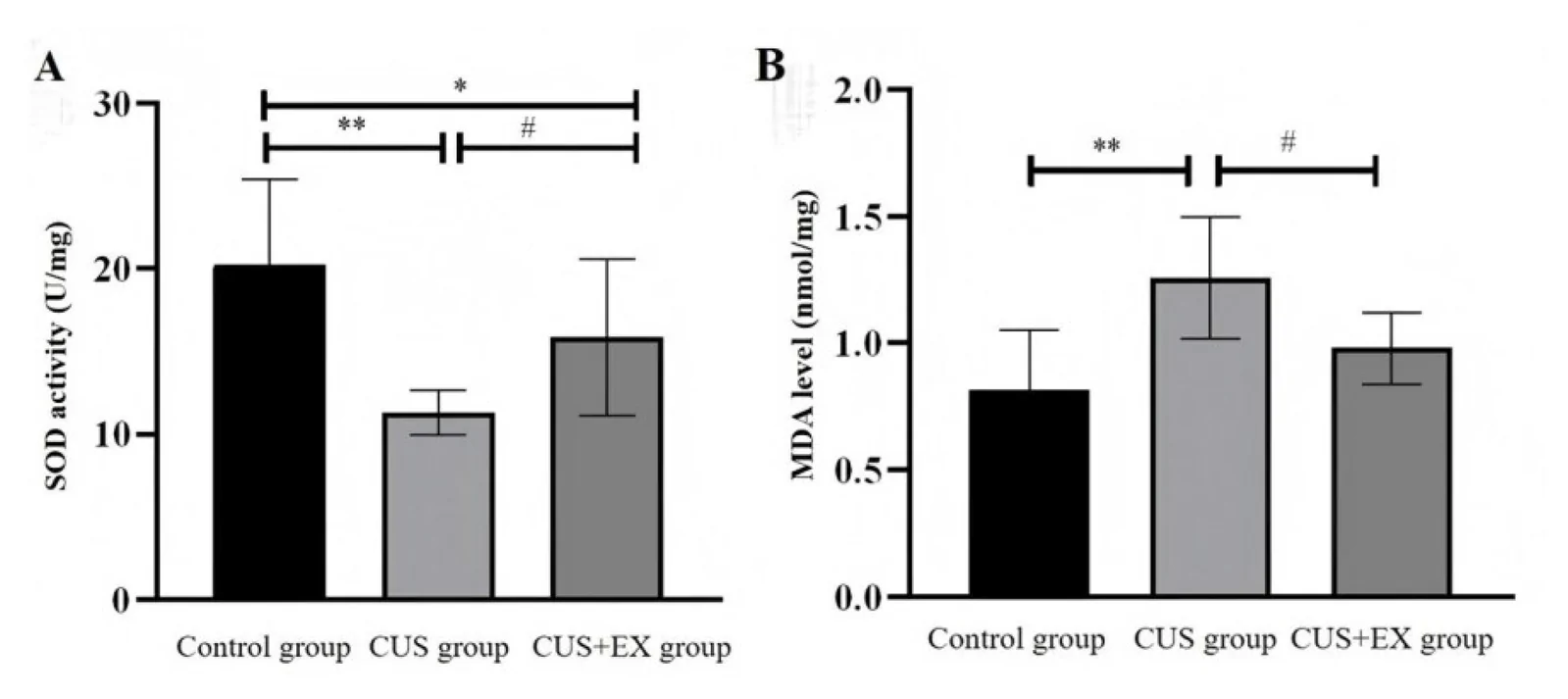
**A,B)** Comparison of plasma SOD activity and MDA level in prefrontal cortex of rats in each group. Data are presented as mean ± standard deviation (SD). n=6 per group. Statistical significance: **P<0.01, *P<0.05 vs control group; #P<0.05, CUS+EX group vs CUS group.

**Fig. 4:**
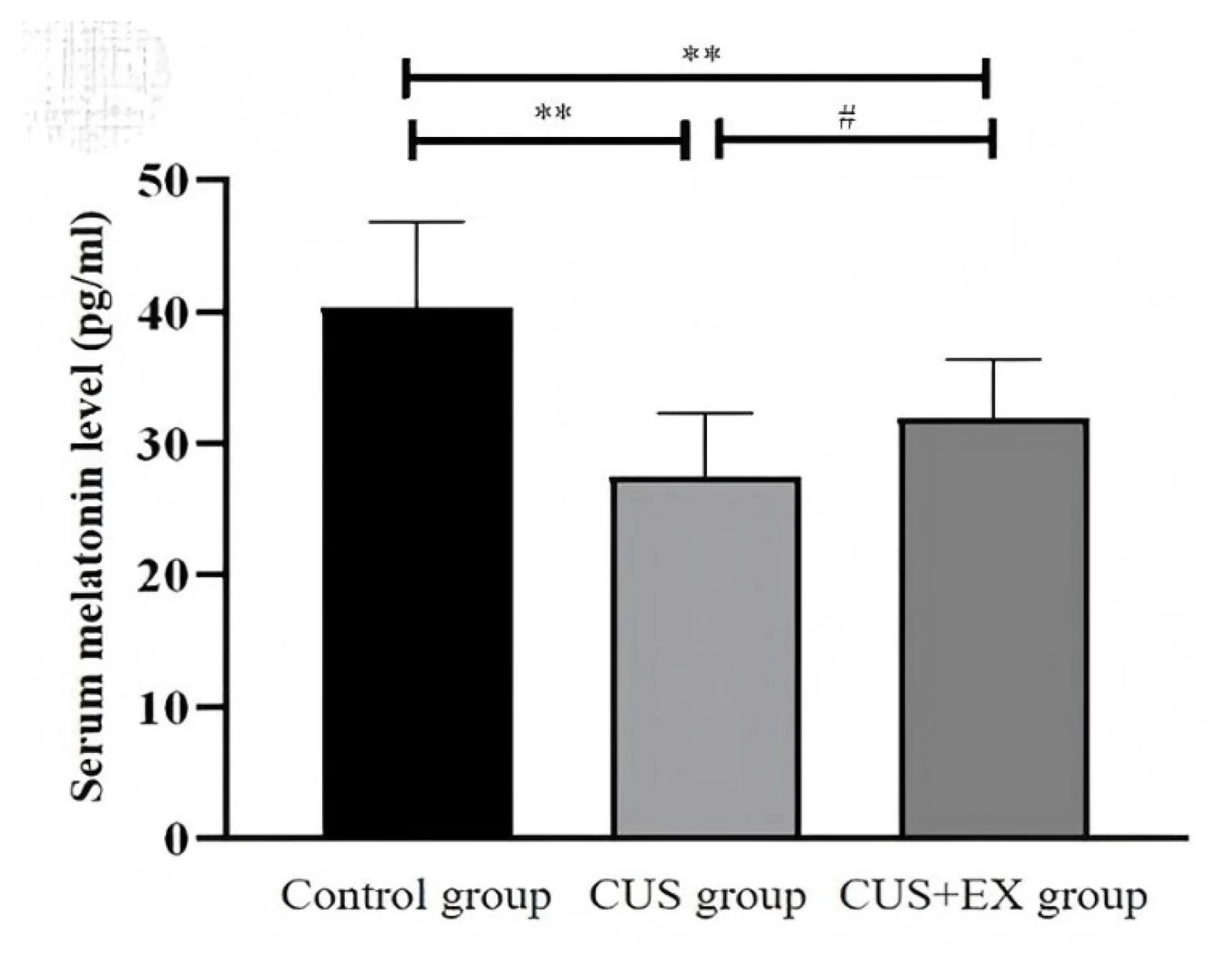
Comparison of plasma melatonin level in each group of rats. Data are presented as mean ± SD. n=12 per group. Statistical significance: **P<0.01, *P<0.05 vs control group; #P<0.05, CUS+EX group vs CUS group.

Compared with the control group, the CUS and CUS+EX groups had significantly lower plasma SOD activity (Fig. 3) and melatonin levels in the PFC (p<0.01, p<0.05) (Fig. 4), whereas MDA levels were significantly higher in the CUS group (p<0.01). After 4 weeks of treadmill exercise, the CUS+EX group had significantly higher plasma melatonin levels and SOD activity and lower MDA levels in the PFC compared with the CUS group (p<0.05). These data indicate that treadmill exercise prevented the CUS-induced decline in melatonin and SOD activity and attenuated the CUS-induced increase in MDA (Fig. 3 and Supplementary Results).

### Protein expression of SIRT1 and Nrf2 in the prefrontal cortex

**Fig. 5:**
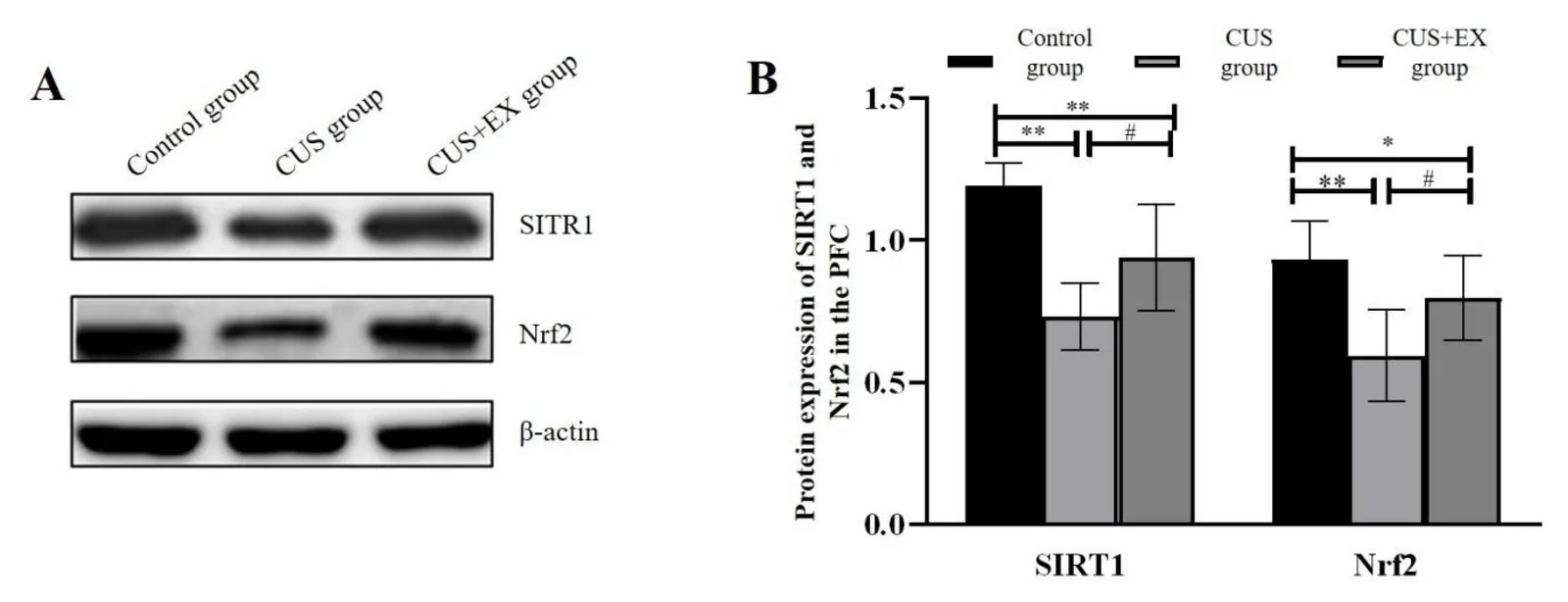
**A,B)** The protein expression of SIRT1 and Nrf2 in the PFC of rats in each group. Data are presented as mean ± standard deviation (SD). n=6 per group. Statistical significance: **P<0.01, *P<0.05 vs control group; #P<0.05, CUS+EX group vs CUS group.

Western blot results showed that the CUS and CUS+EX groups had significantly lower protein expressions of SIRT1 and Nrf2 in the PFC compared with the control group (p<0.01, p<0.05). After 4 weeks of treadmill exercise, the CUS+EX group had significantly higher protein expressions of SIRT1 and Nrf2 in the PFC compared with the CUS group (p<0.05). These findings indicate that treadmill exercise prevented the CUS-induced reduction in SIRT1 and Nrf2 protein expression (Fig. 5).

## Discussion

Studies have shown that almost 80 % of people with depression have circadian rhythm or sleep disturbances, and melatonin plays an antidepressant role by regulating and restoring circadian rhythms [20–22]. As a melatonin receptor agonist, agomelatine can improve major depressive symptoms by advancing sleep stages, lowering body temperature and increasing melatonin secretion. In rat models of depression, melatonin has been shown to induce rapid and long-lasting antidepressant effects [23]. Many studies have confirmed that plasma melatonin levels are significantly reduced in patients with depression [24,25]. In this study, CUS rats showed decreased plasma melatonin levels and depression-like behaviors such as hopelessness, reduced exploratory behavior and decreased motor activity, which are similar to the clinical symptoms of depression, suggesting that depression-like behaviors induced by CUS may be related to decreased plasma melatonin levels.

Although the effect of exercise on melatonin levels is somewhat controversial, previous studies have shown that exercise can improve the sleep quality of children with autism by increasing melatonin levels. However, exercise in dim-light conditions may advance melatonin rhythms without significantly affecting plasma melatonin levels. Nocturnal exercise significantly decreased the level of plasma melatonin at night, increased sleep latency and decreased sleep efficiency. Animal studies have confirmed that moderate intensity exercise during the day (especially in the morning) can increase melatonin secretion, whereas nighttime exercise will reduce its secretion [26–28]. In this study, 4 weeks of treadmill exercise (conducted from 9:00 to 11:00) significantly increased plasma melatonin levels and improved depressive behavior in CUS rats, supporting the hypothesis that melatonin may play a role in exercise-induced improvement in depressive behavior in CUS rats. However, the specific signaling pathway mechanism still needs further study.

The TST and OFT are widely used to assess depressive-like and exploratory/locomotor behaviors in rodents. In the TST, a reduced latency to the first immobility and an increased total immobility time are commonly interpreted as elevations in behavioral despair or learned helplessness. In the OFT, decreased horizontal crossings, fewer rearings and reduced time spent in the central area reflect diminished spontaneous locomotion and exploratory drive, which can manifest as depressive-like or behavioral inhibition. In the present study, CUS prevented TST immobility and decreased OFT exploratory measures, whereas treadmill exercise partially reversed these changes. These behavioral results are therefore consistent with an antidepressant-like effect of exercise and provide behavioral context for the observed changes in plasma melatonin, PFC oxidative stress markers (SOD, MDA) and SIRT1/Nrf2 signaling.

Oxidative stress is an important aspect of depression’s pathogenesis, which is characterized by the increase of free radical production and the decrease of antioxidant activity, resulting in an imbalance between oxidation and antioxidants and subsequent neuron damage through lipid peroxidation [29]. The PFC is highly sensitive to oxidative stress and plays a key role in CUS-induced depression. Superoxide dismutase and MDA are commonly used oxidative stress indicators [30]. Here, consistent with previous studies, CUS significantly reduced SOD activity and prevented MDA levels in the PFC, suggesting that PFC oxidative stress is directly involved in the development of depression.

Sirtuin 1 is an NAD-dependent Class III histone deacetylase that regulates cell aging, inhibits apoptosis, reduces inflammation and mediates antioxidant responses. It plays an important role in the pathogenesis and treatment of neuropsychiatric diseases [31]. A recent genome-wide association study found that a locus in the SIRT1 gene was associated with major depression, and lower SIRT1 mRNA levels were observed in the blood of patients with depression. Studies have shown that SIRT1 mRNA expression levels are inversely correlated with the severity of depression, with lower SIRT1 mRNA levels associated with higher Hamilton depression scores. Animal studies have demonstrated that chronic stress reduces SIRT1 activity in the dentate gyrus of the hippocampus, and inhibition of SIRT1 function by pharmacological or genetic means increases depression-like behavior in mice [32]. In contrast, injection of SIRT1 activators produces antidepressant effects and improves abnormal dendritic structural development induced by chronic stress exposure. These results suggest that SIRT1 plays an important role in the pathogenesis of depression.

Furthermore, Nrf2 is a key regulator of oxidative stress response and an important downstream target of SIRT1. The function of SIRT1 is largely dependent on the activation of Nrf2, which is transferred from the cytoplasm to the nucleus and activates the transcription of multiple genes to enhance the antioxidant response and reduce sensitivity to oxidative stress [33]. Recent reports suggest that the SIRT1/Nrf2 pathway is strongly associated with depression. Chronic social frustration stress induced anxiety, depression-like behavior and cognitive impairment in male C57BL/6 mice by down-regulating SIRT1/Nrf2 protein expression in the hippocampus [34]. Since SIRT1 and Nrf2 are heavily expressed in brain regions such as the hippocampus and PFC, we focused on the PFC in this study. Our results showed that 4-week CUS significantly reduced SIRT1 and Nrf2 protein expression in the PFC. Therefore, the depression-like behavior induced by CUS in rats may be related to the inhibition of SIRT1 expression in the PFC, the increase of Nrf2 binding to its specific receptor and the decrease of Nrf2 activity, which leads to the enhancement of oxidative stress in the PFC (decrease of SOD activity and increase of MDA level). Further research is needed to clarify the specific mechanisms.

Considering that aerobic exercise can effectively regulate negative emotions and has been shown to improve CUS-induced depressive behavior by reducing oxidative stress damage in the brain, this study found that 4 weeks of treadmill exercise significantly prevented SOD activity and reduced MDA levels in the PFC of CUS rats [35,36]. This suggests that aerobic exercise can improve depressive behavior in CUS rats by enhancing the antioxidant capacity of the PFC. The Nrf2 transcription factor is a key aspect in inducing a variety of antioxidants and plays a key role in reducing CUS-induced oxidative damage in brain tissue [37]. Under normal circumstances, Nrf2 is located in the cytoplasm, but under oxidative stress, it transfers to the nucleus and binds to antioxidant response elements in target gene promoters, inducing transcription of multiple genes, thereby enhancing antioxidant response and reducing sensitivity to oxidative stress [38]. There is increasing evidence that SIRT1 can enhance the expression and transcriptional activity of Nrf2, thereby exerting a powerful antioxidant effect. In addition, exercise has been shown to up-regulate SIRT1, activate Nrf2 and promote increases in brain-derived neurotrophic factor and cAMP response element binding protein, and improve synaptic plasticity and neurogenesis, and thus alleviate depression. This study found that 4 weeks of treadmill exercise significantly prevented SIRT1 and Nrf2 protein expression in the PFC in CUS rats, suggesting that exercise may improve depression-like behavior by activating SIRT1/Nrf2 signaling pathways in the PFC and reducing oxidative stress.

Melatonin is a powerful antioxidant that scavenges free radicals, reduces oxidative stress damage and suppresses neuroinflammation. Recent studies have shown that melatonin can protect the hippocampal dentate gyrus from lipopolysaccharide-induced oxidative stress damage, acute neuroinflammation and apoptotic neurodegeneration in rats up to 7 days after birth by activating the SIRT1/Nrf2 signaling pathway [39–41].

Although this study provides evidence that treadmill exercise ameliorates depressive-like behaviors in CUS rats potentially via modulation of melatonin levels and SIRT1/Nrf2 signaling in the PFC, several important limitations should be acknowledged.

Causality and mechanistic specificity. The proposed pathway (Exercise → Melatonin → SIRT1/Nrf2 → Antioxidant defense) remains correlational. Without pharmacological blockers (e.g. melatonin receptor antagonists luzindole/4-P-PDOT, or SIRT1 inhibitors EX-527/sirtinol) or genetic manipulations, we cannot confirm that exercise-induced benefits are specifically dependent on this pathway rather than associated with it. Future studies incorporating these interventions are needed to establish causal necessity and sufficiency.

Sex and circadian limitations. The study included only male rats, limiting generalizability to females given known sex differences in stress responses and depression mechanisms. Additionally, morning exercise (09:00–11:00) may have phase-shifted melatonin rhythms; without time-of-day controls or 24-hour profiling, we cannot exclude circadian confounds from exercise-specific effects.

Other limitations. Our analyses were restricted to the PFC, whereas depression involves multiple brain regions. Downstream molecular mechanisms and alternative pathways (neuroinflammation, neurogenesis) were not explored. Sample sizes (n=12 for behavior, n=6 for biochemistry) may limit detection of small effects. The three-group design demonstrates associations but cannot establish causality without pharmacologic controls or dose–response assessments.

## Conclusions

These findings demonstrate that treadmill exercise is associated with increased melatonin levels, enhanced SIRT1/Nrf2 pathway activation, reduced oxidative stress, and improved depressive-like behaviors in CUS rats. Even though these results are consistent with the hypothesis that melatonin-SIRT1/Nrf2 signaling contributes to exercise-induced antidepressant effects, the present correlational design does not establish causality. Direct mechanistic evidence is still needed and awaits future studies incorporating pharmacological or genetic manipulation of this pathway.

## Supplementary Information
